# Non-Descemet Stripping Automated Endothelial Keratoplasty (nDSAEK) for Late Endothelial Failure After Mushroom Keratoplasty: A Retrospective Analysis of Visual and Anatomical Outcomes

**DOI:** 10.3390/jcm14155568

**Published:** 2025-08-07

**Authors:** Antonio Moramarco, Natalie di Geronimo, Marian Sergiu Zimbru, Arianna Grendele, Francesco Biagini, Maurizio Mete, Vito Romano, Luigi Fontana

**Affiliations:** 1 Ophthalmology Unit, Dipartimento di Scienze Mediche e Chirurgiche, Alma Mater Studiorum University of Bologna, 40126 Bologna, Italy; natalie.digeronimo@outlook.it (N.d.G.); marianzimbru@gmail.com (M.S.Z.); maumete@gmail.com (M.M.); luifonta@gmail.com (L.F.); 2 IRCCS Azienda Ospedaliero-Universitaria di Bologna, 40138 Bologna, Italy; 3 Studio Oculistico d’Azeglio, 40123 Bologna, Italy; 4 Ophthalmology Unit, Ospedale Morgagni-Pierantoni, 47121 Forlì, Italy; francesco.biagini1994@gmail.com; 5 Eye Unit, Department of Medical and Surgical Specialties, Radiological Sciences, and Public Health, University of Brescia, 25121 Brescia, Italy; vito.romano@gmail.com

**Keywords:** mushroom penetrating keratoplasty, late endothelial failure, non-Descemet stripping automated endothelial keratoplasty, nDSAEK, DSAEK-on-PK

## Abstract

**Background**: Mushroom penetrating keratoplasty (MPK) is an alternative to traditional penetrating keratoplasty (PK) that offers improved graft survival and reduced immunological rejection. However, MPK grafts may still experience endothelial failure over time. This study evaluates the outcomes of non-Descemet Stripping Automated Endothelial Keratoplasty (nDSAEK) as a surgical approach for endothelial decompensation following MPK. **Methods**: A monocentric, retrospective study was conducted at the Ophthalmology Department of Sant’Orsola-Malpighi Hospital, including patients who underwent nDSAEK for endothelial failure after MPK between 2022 and 2024. Pre- and postoperative best-corrected visual acuity (BCVA), central corneal thickness (CCT), and endothelial cell density (ECD) were assessed. **Results**: Eighteen eyes from 18 patients (mean age: 39.94 years) were included. Primary MPK indications were post-keratitis leucoma (77.7%), traumatic scarring (16.7%), and keratoconus (5.6%). At one year, mean BCVA improved significantly from 1.40 ± 0.42 logMAR to 0.46 ± 0.19 logMAR (*p* < 0.05), and mean CCT decreased from 721 ± 70.12 µm to 616 ± 52.80 µm (*p* < 0.05). The mean postoperative ECD was 1748 ± 100 cells/mm^2^, with lower eye values requiring re-bubbling. No immunological rejection or graft failures were reported. **Conclusions**: nDSAEK is a promising treatment for MPK endothelial failure, demonstrating good visual and anatomical outcomes.

## 1. Introduction

Mushroom penetrating keratoplasty (MPK) is an emerging alternative to traditional penetrating keratoplasty (PK), progressively gaining popularity. This technique and its main advantages were first described by Busin in 2005 [1]. In MPK, two distinct corneal grafts are transplanted from the same donor: a large, superficial button (the “hat”) and, beneath it, a smaller, deep button (the “stem”). This way, the anterior stromal lamella can have a larger diameter than the traditionally used diameters in PK, thereby reducing postoperative astigmatism without increasing the risk of graft rejection. This is achieved by preserving a more significant portion of the recipient’s endothelium, because of the use of an inner lamella with a smaller diameter than the first, allowing for the selective replacement of the pathological endothelium only along the visual axis, consequently minimizing the local immune response towards the donor [1,2,3,4].

In traditional PK, the leading cause of graft failure is late endothelial failure without any history of immunological reaction [5,6]. The progressive decrease in endothelial cell function over time can eventually result in endothelial decompensation and visually significant corneal edema. The graft’s survival time is notably shortened when keratoplasty is performed in eyes with pre-existing endothelial dysfunction (e.g., bullous keratopathy) compared to those with healthy recipient endothelium (e.g., stromal scarring or keratoconus) [7,8,9]. Additionally, repeated keratoplasties are associated with reduced graft survival time and increased risk of immunological rejection due to heightened allosensitization [10,11]. Furthermore, additional surgical procedures, such as phacoemulsification and vitrectomy, may further exacerbate endothelial cell density (ECD) loss and consequently corneal central thickness (CCT) [12]. The corneal endothelium is a single layer of hexagonal cells on the inner surface of the cornea that maintains corneal transparency by actively regulating fluid transport; this function is essential for preserving normal corneal thickness, as excess fluid accumulation leads to increased thickness and loss of transparency. Central corneal thickness is defined as the distance between the anterior surface of the epithelium and the posterior surface of the endothelial layer. It does not represent the thickness of the entire cornea as this structure shows a gradually increasing thickness from the center to the periphery. It is known that the average CCT is approximately 550 μm, and this thickness can be around 23% greater in the peripheral cornea [13]. Zaman concluded that the mean CCT of 230 different data sets involving measurements of >14,000 individuals was 536 μm [14]. In healthy adults aged 20–39 years, the average endothelial cell density is around 3000 cells/mm^2^, persistently diminishing with age, maintaining an average decline of around 0.3–0.6% per year. Under normal circumstances, endothelial cell density reaches an average of 2600 cells/mm^2^ in healthy adults aged 60–79 years [15].

Overall, MPK grafts tend to demonstrate greater longevity than traditional PK grafts, as the rate of ECD loss is slower, and immunological rejection is less frequent due to the smaller size of the endothelial area replaced [5,6]. Other factors can determine the failure of an MPK graft, such as persistent epithelial defect with anterior lamellar melting or reactivation of herpetic or other infective diseases [16].

Surgical treatment of endothelial decompensation poses a significant challenge in MPK and traditional PK. Options include a repeat penetrating keratoplasty or an endothelial keratoplasty (EK) and either Descemet Membrane Endothelial Keratoplasty (DMEK) or Descemet Stripping Automated Endothelial Keratoplasty (DSAEK). In DSAEK, the graft consists of endothelium, Descemet membrane, and a thin layer of stroma, whereas a DMEK graft includes only the endothelium and Descemet membrane. EK offers several advantages over PK, including faster visual rehabilitation, reduced induced astigmatism, and lower risk of rejection [17]. However, when performed as a secondary procedure for failed PK, EK is also associated with a higher primary postoperative failure rate and an additional risk of complications, mainly graft dislocation [6,17,18]. Among EK techniques, DMEK for failed PK demonstrates better long-term visual outcomes but is associated with a higher graft detachment rate [8]. This is consistent with the outcomes described for primary EK. In contrast, although DSAEK generally yields slightly inferior visual outcomes compared to DMEK, it remains technically easier to perform in complex eyes and carries a lower risk of graft detachment [19,20,21].

While performing EK over a failed penetrating keratoplasty, careful attention must be given during the descemetorhexis phase, as it may cause disruption of the graft–host interface and create significant posterior surface irregularity, thereby increasing the risk of complications such as graft dislocation or suboptimal visual outcomes. Interestingly, evidence suggests that both DMEK and DSAEK can be performed without descemetorhexis. In such cases, the EK graft is positioned directly on the failing endothelium without compromising long-term best-corrected visual acuity (BCVA) and ECD [21,22,23,24,25]. For DSAEK, this technique is commonly referred to in the literature as non-Descemet Stripping Automated Endothelial Keratoplasty (nDSAEK) [24,26].

Despite the growing use of DSAEK and DMEK for treating primary endothelial disease and failure following PK, there is a noticeable gap in the literature regarding their application in the context of endothelial decompensation following MPK. This lack of data highlights the need for focused research to understand better the outcomes, feasibility, and potential challenges of EK procedures in this surgical setting.

Our current work aims to address this gap in the literature by evaluating the outcomes of non-Descemet Stripping Automated Endothelial Keratoplasty (nDSAEK) as a surgical approach for managing endothelial dysfunction in failed mushroom penetrating keratoplasty (MPK).

## 2. Materials and Methods

### 2.1. Patient Selection

This monocentric, retrospective study was conducted at the Ophthalmology Department of Sant’Orsola-Malpighi Hospital in Bologna. It included eyes that underwent nDSAEK procedures for endothelial decompensation following primary MPK, with or without bullous keratopathy, and presented to our clinic between 2022 and 2024.

The following exclusion criteria were considered: amblyopia, glaucoma or uncontrolled intraocular pressure, history of retinal detachment or previous retinal surgery, age-related macular degeneration (AMD), significant cataracts, severe ocular surface disease (e.g., Steven–Johnson syndrome or ocular cicatricial pemphigoid), presence of systemic conditions that may compromise corneal outcomes (e.g., uncontrolled diabetes), poor compliance or inability to adhere to follow-up schedules.

### 2.2. Clinical Evaluation and Measurements

Clinical evaluation included best-corrected visual acuity (BCVA), slit-lamp examination (SLE), central corneal thickness (CCT) measured by Scheimpflug corneal tomography (Pentacam, Oculus Optikgeraete GmbH; Wetzlar, Germany), and ECD measured by specular microscopy (EM-400, Tomey^®^, Nagoya, Japan. Software version: 1F.1U). The postoperative assessment included anterior segment OCT (CASIA2, Tomey^®^, Nagoya, Japan. Software version: 50.6A.02). Rejection episodes, survival rate, and postoperative complications were recorded.

**Figure 1:**
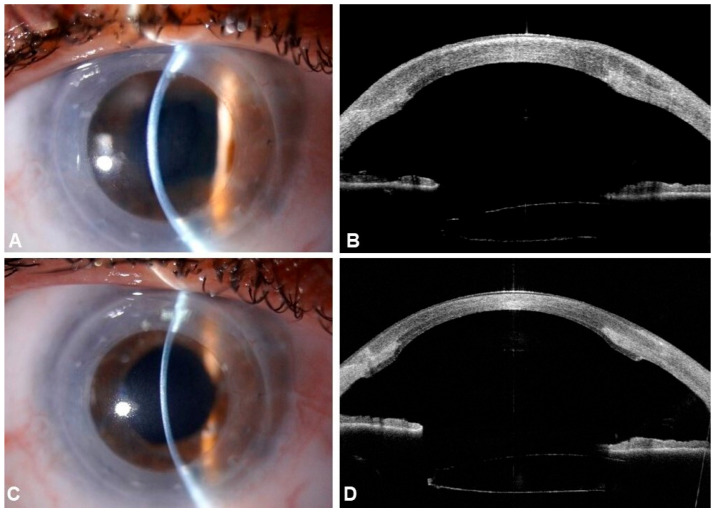
(**A**,**B**) Anterior segment photographs and anterior segment OCT of one of our cases preoperatively and (**C**,**D**) at 12-month follow-up postoperatively, showing marked improvement in corneal edema and clarity.

Routine clinical and instrumental examinations were scheduled at 1 and 7 days and 1, 2, 6, and 12 months after the procedure. Representative anterior segment photographs and OCT images from the preoperative period and the 12-month follow-up are presented in Figure 1.

The primary endpoint was postoperative BCVA (measured in a LogMAR scale at a distance). Postoperative CCT and ECD were considered secondary outcomes. All measures were obtained at one year or at the last follow-up visit.

### 2.3. nDSAEK Procedure

All nDSAEK procedures on MPK were performed by experienced cornea surgeons (L.F. and A.M.) at our hospital. Patients underwent local peribulbar anesthesia and received no routine preoperative antibiotic or anti-inflammatory treatment. The Corneal Bank of Bologna provided donor corneas.

Donor DSAEK grafts were initially prepared on-site with a single-use blade microkeratome (Moria^®^ One Use-Plus System, Antony, France) before the procedure. Donor thickness was measured with intraoperative OCT (Leica Proveo 8, Leica Microsystems, Wetzlar, Germany), and a target graft thickness of around 120 µm was chosen.

Side-ports and a main incision were made using a 1.1 mm and 2.4 mm keratome, respectively. No Descemet–endothelium stripping was performed on the original graft. All patients presented with an inferior iridectomy from the primary procedure.

Final trephination of the DSAEK graft was completed on the surgical table with an 8 mm diameter punch (Moria SA, Antony, France), and graft insertion was performed via the main incision with a pull-through technique following enlargement of the main tunnel using a 2.75 mm blade. After ensuring accurate centration, 20% sulfur hexafluoride (SF6) gas was injected into the anterior chamber to stabilize the graft. In phakic patients, an air bubble was injected instead. Throughout the procedure, intraoperative OCT was used as a guide for correctly positioning the flap, especially in cases of poor visibility [27].

Postoperatively, patients underwent SLE two hours after the procedure. If excessive gas was observed, partial evacuation through the side port was performed with a 30 Ga needle. Patients were instructed to maintain a supine posture in the days following the surgery to maximize the effect of the gas bubble.

All patients received postoperative topical betamethasone 0.13%/chloramphenicol 0.25% association, which was slowly tapered over two months, and topical sodium bromfenac twice daily during the first month postoperatively. No systemic postoperative medications were prescribed.

### 2.4. Re-Bubbling

Graft re-bubbling was performed in case of significant DSAEK graft detachment in the first seven postoperative days. Significant detachment was defined as more than 33% of the graft surface and/or central detachment, detected at SLE, and confirmed with anterior segment OCT. This procedure was performed in the operating room by injecting 20% SF6 or air into the anterior chamber under topical anesthesia. Patients were instructed to maintain a supine position and were re-examined the following day.

### 2.5. Statistical Analysis

Descriptive variables are presented as mean and standard deviation (SD). The normal distribution of the variables was verified using the Shapiro–Wilk test for each variable. A two-sided paired Student *t*-test was performed to compare preoperative and postoperative measurements. *p*-values < 0.05 were considered statistically significant. All statistical analyses were performed using InStat software (version 3.10) and Microsoft Excel (Microsoft Corp, Redmond, WA, USA, version 16.93.1).

## 3. Results

The study included 18 eyes from 18 patients who underwent nDSAEK after the failure of a primary MPK. Of these, 8 were men and 10 were women, with a mean age of 39.94 ± 13.46 years. The primary indications for MPK were post-keratitis leucoma (14 cases, 77.7%), traumatic corneal scar (3 cases, 16.7%), and advanced keratoconus (1 case, 5.6%). Subsequent DSAEK surgery was performed due to endothelial rejection in 1 case (7%) and late graft failure in 14 cases (93%). No significant visual comorbidities were noted. The mean interval between MPK and nDSAEK was 37.2 ± 23.9 months. The average follow-up period was 18.6 months, with a minimum follow-up of 12 months in all cases. A summary of patient characteristics, including demographics, indications for MPK, and interval between MPK and nDSAEK, is presented in Table 1.

Re-bubbling was required in 2 eyes (11.1%), each requiring a single re-bubbling procedure.

Preoperatively, the mean BCVA was 1.40 ± 0.42 logMAR, which significantly improved in all patients at each follow-up visit, with a mean BCVA of 0.57 ± 0.23 logMAR (*p* < 0.05) at three months, 0.48 ± 0.19 logMAR (*p* < 0.05) at six months, and 0.46 ± 0.19 logMAR (*p* < 0.05) at one year.

Mean preoperative CCT was 721 ± 70.12 µm, which significantly decreased postoperatively to 624 ± 45.97 µm (*p* < 0.05) at three months, 611 ± 50.43 µm (*p* < 0.05) at six months, and 616 ± 52.80 µm (*p* < 0.05) at one year.

**Figure 2:**
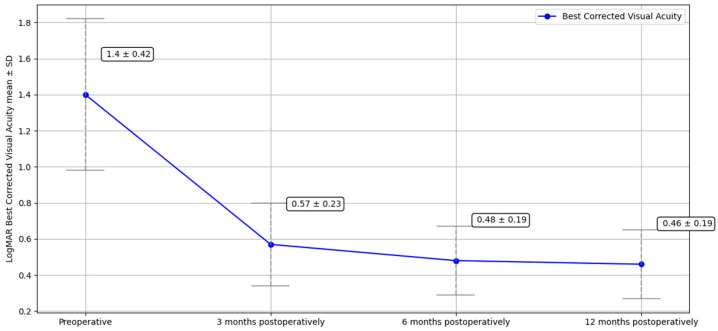
Best-corrected visual acuity (BCVA) preoperatively and at 3, 6, and 12 months following nDSAEK on MPK.

**Figure 3:**
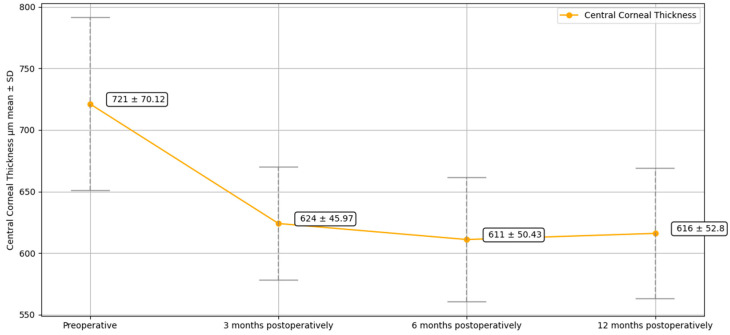
Central Corneal Thickness (CCT) preoperatively and at 3, 6, and 12 months following nDSAEK on MPK.

**Figure 4:**
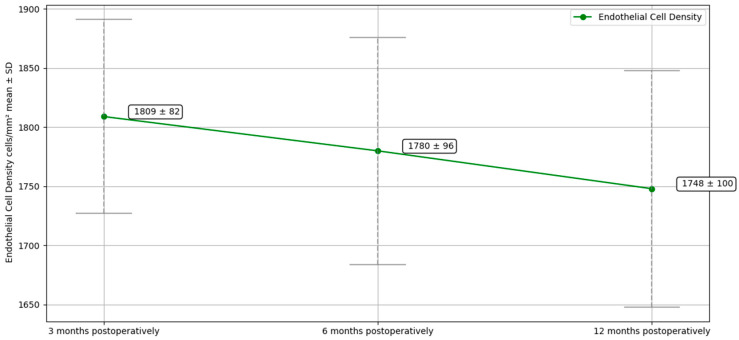
Endothelial Cell Density (ECD) at 3, 6, and 12 months following nDSAEK on MPK.

Preoperative ECD could not be measured in all patients because readings in edematous corneas were unreliable. However, the overall mean postoperative ECD was 1809 ± 82 cells/mm^2^ at three months, 1780 ± 96 cells/mm^2^ at six months, and 1748 ± 100 cells/mm^2^ at one year. The decrease in ECD during the follow-up was not statistically significant. Preoperative and follow-up data at 3, 6, and 12 months for BCVA, CCT, and ECD is summarized in Table 2 and shown in Figure 2, Figure 3, and Figure 4, respectively.

**Figure 5:**
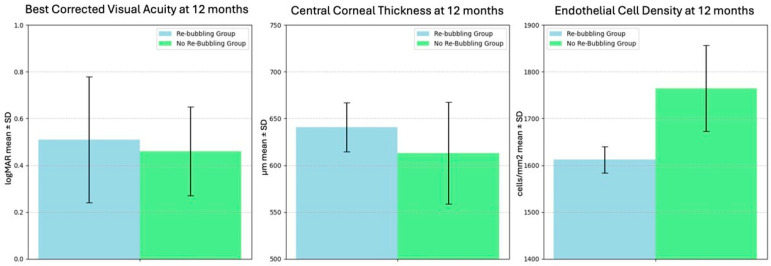
Comparison of best-corrected visual acuity (BCVA), central corneal thickness (CCT), and endothelial cell density (ECD) at 12 months postoperatively between the re-bubbling group and the no re-bubbling group.

In eyes that did not require re-bubbling, the mean postoperative ECD at one year was 1765 ± 92 cells/mm^2^, whereas in eyes requiring re-bubbling, the mean ECD was 1612 ± 28 cells/mm^2^. The mean BCVA was 0.51 ± 0.27 LogMAR in the re-bubbling group and 0.46 ± 0.19 LogMAR in the no re-bubbling group, and CCT was 641 ± 26.16 µm and 613 ± 54.94 µm, respectively. The primary and secondary outcomes differences between the two groups at baseline and at 12 months are summarized in Table 3 and Figure 5.

No intraoperative complications were observed. During the entire follow-up period, no cases of immunological graft rejection or need for repeat nDSAEK due to graft failure were reported.

## 4. Discussion

The global trend in corneal transplantation has progressively shifted from full-thickness penetrating keratoplasty (PK) to more selective and less invasive lamellar techniques. In the United States, endothelial keratoplasty (EK) procedures have surpassed penetrating keratoplasty in annual volume as early as 2012, and this shift has only continued to accelerate in subsequent years, particularly in the treatment of endothelial decompensation [28,29]. This transition is primarily driven by the significant advantages offered by lamellar approaches, including reduced surgical invasiveness, faster and more predictable visual recovery, improved postoperative visual acuity, and a lower incidence of immune-mediated rejection. Among EK procedures, Descemet Stripping Automated Endothelial Keratoplasty (DSAEK) and Descemet Membrane Endothelial Keratoplasty (DMEK) have become standard techniques, with DMEK offering the most anatomically selective approach by replacing only the diseased endothelium and Descemet’s membrane [30]. On the other hand, for stromal pathologies that do not involve the entire corneal thickness, deep anterior lamellar keratoplasty (DALK) has gained considerable popularity, especially following refinements in surgical technique such as the introduction of the “big bubble” method. These advances have enabled better and more consistent separation of the Descemet’s membrane from the overlying stroma, making DALK a viable and often preferable alternative to PK in many non-endothelial disorders. As a result, penetrating keratoplasty has gradually become a niche procedure, typically reserved for complex cases where full-thickness stromal involvement is present and where lamellar approaches such as DALK are either not feasible or have failed. These include advanced keratoconus with deep stromal scarring or full-thickness leucomas, sequelae of infectious keratitis with structural compromise, and severe traumatic injuries resulting in corneal perforation [29]. Even in keratoconus, the historical leading indication for penetrating keratoplasty, the trend has shifted. The widespread adoption of corneal collagen cross-linking (CXL) has proven effective in halting disease progression, thereby delaying or even avoiding the need for surgical intervention. Furthermore, advancements in contact lens technologies, including rigid gas-permeable (RGP) lenses, scleral lenses, and piggy-back systems, have significantly improved the visual rehabilitation of patients with irregular astigmatism or advanced ectasia, often deferring or replacing the need for surgical intervention [31,32].

MPK is indicated in cases of diseases affecting the full thickness of the cornea, especially in the central portion. Primary examples include advanced keratoconus with stromal opacification, corneal scarring resulting from infections or trauma, or corneal dystrophies. Lamellar keratoplasty (LK), such as deep anterior lamellar keratoplasty (DALK), is a practical approach to treating superficial stroma disease and ectasia while ultimately preserving the host endothelium and avoiding complications associated with an open-sky procedure [33]. However, anterior LK is unsuitable when there is a full-thickness corneal involvement, particularly in the central optical zone [16,34].

Due to the smaller proportion of total endothelial cells transplanted, MPK grafts tend to last longer than traditional PK grafts [4,6]. However, over time, eyes that have undergone MPK may still develop endothelial failure and visually significant corneal edema due to progressive endothelial cell loss, with or without associated immunological reaction. For endothelial failure in traditional PK, numerous studies have illustrated the efficacy of repeat PK and EK, including DMEK and DSAEK, in restoring corneal clarity and visual function [6,17,18,19,23,24,35,36]. However, evidence regarding the surgical management of endothelial failure in MPK remains limited, likely due to its relatively recent adoption as a surgical technique. Most endothelial failure in traditional PK is typically observed after at least 10–15 years postoperatively, and MPK was described only in 2005 [1,5,8].

Among the 18 patients included, the primary indications for MPK were post-keratitis corneal leucoma (77.7%), traumatic corneal scarring (16.7%), and advanced keratoconus (5.6%). Graft failure was primarily attributed to endothelial graft failure without signs of immunological rejection (93%) and, in one case, to immunologic endothelial rejection (7%).

The mean age of the patients included in our study was 39.9 years. This highlights MPK’s role in conditions typically affecting younger individuals, such as keratoconus, trauma, and infectious keratitis. In contrast, the mean age for PK reported in the literature ranges between 50 and 60 years. However, this trend is expected to decline, as EK has become the preferred technique for treating bullous keratopathy and endothelial dystrophies, gradually reducing the indications for PK in older patients [28,29,37].

Overall, EK shows several advantages over repeat PK, including reduced invasiveness, faster visual rehabilitation, reduced induced astigmatism, and lower risk of rejection [6,17,35]. Compared to DMEK, DSAEK is a more forgiving procedure, avoiding the challenges of graft unfolding and orientation challenges under an opaque cornea, with DSAEK grafts exhibiting a lower graft detachment rate [19,20]. Additionally, as first performed by Price in 2006, DSAEK can be performed without prior descemetorhexis while maintaining long-term BCVA and ECD [19,25,38]. Indeed, in non-Fuchs bullous keratopathy, Descemet’s membrane remains intact, and it has no guttae that may interfere with vision after endothelial keratoplasty. The reduction in visual acuity results from corneal edema secondary to endothelial cell loss. Consequently, removal of Descemet’s membrane may not be required [24]. This is especially useful in eyes that have undergone MPK, as Descemet membrane stripping may cause trauma and weaken the mechanical strength at the junction between the deeper endothelial button and the recipient cornea, potentially compromising the structural integrity of the posterior surface of the cornea and likely affecting the adhesion of the new endothelial graft. Thus, this study aims to evaluate the efficacy and safety of DSAEK without Descemet membrane stripping as a surgical treatment for endothelial failure following MPK.

Initially, there were concerns regarding visual acuity outcomes and interface quality following nDSAEK. However, several studies have confirmed the non-inferiority of nDSAEK compared to DSAEK and the good quality of the interface [24,39,40]. Omoto et al. conducted a five-year follow-up analysis of the outcomes in 28 nDSAEK and 22 DSAEK cases. No statistically significant differences were observed between nDSAEK and DSAEK eyes regarding changes in ECD or BCVA at any time point [39]. Moreover, Kamiya et al. analyzed the effect of intraocular forward scattering or high-order aberrations after the two techniques, and their findings revealed no significant differences between the DSAEK and nDSAEK subgroups in postoperative OSI, corneal HOAs, logMAR UCVA, or logMAR BSCVA. This suggests that the presence or absence of Descemet’s membrane does not substantially impact visual acuity, higher-order aberrations, or intraocular forward scattering after surgery. A possible explanation is that Descemet’s membrane is a thin, morphologically uniform structure, minimizing its influence on optical quality [40].

Regarding interface quality, analyses were performed using anterior segment OCT and confocal microscopy to assess potential differences between these techniques and the presence of opacities that could compromise visual quality. After nDSAEK, the residual host Descemet’s membrane could not be identified using slit-lamp biomicroscopy, whereas in DSAEK cases, the descemetorhexis margin was visible. Similarly, anterior segment OCT did not reveal appreciable differences in the donor–host interface between the two procedures [41]. However, confocal microscopy consistently detected highly reflective particles of varying sizes at the donor–recipient interface following nDSAEK. Similarly to DSAEK, the mean density of these particles decreased significantly over time. A distinguishing feature of nDSAEK was the presence of hyperreflective giant particles exceeding 30 μm in size. Histological analysis suggests that, unlike DSAEK, the primary structural difference in the cornea after nDSAEK is the retention of Descemet’s membrane and compressed host endothelial cells. The authors hypothesize that the observed hyperreflective giant particles may represent necrotic and aggregated host endothelial cells [41].

Another notable finding was the persistence of needle-shaped materials in the mid-to-deep corneal stroma six months after both nDSAEK and DSAEK. In a previous confocal study on DSAEK [42], similar needle-shaped materials were observed in the host stroma preoperatively and postoperatively, though their quantity was not analyzed. Notably, the donor corneal stroma carrying Descemet’s membrane with a healthy endothelium exhibited normal, non-activated keratocytes without needle-shaped materials. Therefore, the presence of these structures, as detected by in vivo laser confocal microscopy, may serve as a marker of the recipient stroma. While these deposits’ origin and clinical significance remain unclear, previous studies suggest they may represent crystalline or lipofuscin accumulations. However, none of these structures determined the development of subepithelial haze or host–recipient interface haze.

Our results are in line with the literature, since the procedure achieved good visual results. The mean BVCA improved from 1.40 ± 0.42 logMAR preoperatively to 0.46 ± 0.19 logMAR at 1 year (*p* < 0.05). On a similar note, mean CCT, another valuable indicator of surgical success, decreased from 721 (±70.12) µm preoperatively to 616 (±52.80) µm at 1 year (*p* < 0.05). This is consistent with the improvement in BCVA, as a decrease in CCT is associated with a reduction in corneal edema, leading to improved visual function.

We observed two cases of postoperative graft detachment that underwent a re-bubbling procedure. The detachment rate in our study was 11%, consistent with reported rates for DSAEK in failed PK (10–20%) and higher than the rate for primary DSAEK (as low as 1.3%) [6,43]. One might expect an increased postoperative donor graft dislocation rate after nDSAEK since the host endothelium is not removed and might be functioning to detach the donor graft. However, a previous study demonstrated that the rate of detachment after nDSAEK has not increased [44]. Omoto et al. described a few cases of graft detachment after nDSAEK, all of which occurred during the first postoperative week, and late graft detachment did not occur in any eyes [39].

The mean postoperative ECD was 1748 ± 100 cells/mm^2^ overall, and it was significantly higher in patients who did not require re-bubbling (1765 ± 92 cells/mm^2^) compared to those who did (1612 ± 28 cells/mm^2^; *p* < 0.05). However, since the subgroup of patients requiring re-bubbling was limited to only two cases, no inferential statistics can be considered reliable. Nevertheless, this suggests that re-bubbling might contribute to a slight reduction in postoperative ECD due to the additional trauma to the endothelial cells. Despite this, the postoperative ECD was deemed acceptable even in case of re-bubbling, reinforced by the fact that all patients demonstrated a good endothelial function at the final follow-up. Instead, no statistically significant difference was found between the two groups regarding BCVA and CCT at 12 months. Omoto et al. found that graft survival rates were worse in the nDSAEK group than in the DSAEK over time, but this difference was not statistically significant. In their study, primary graft failure occurred in 5 eyes in the nDSAEK group and 3 in the DSAEK group. However, multivariate analysis did not show the selection of nDSAEK or DSAEK as a significant risk factor for graft failure. The endothelial cell loss rate after nDSAEK reported in this study (22% cell loss after 6 months and 29% cell loss after 1 year) was again comparable to that reported so far: 34% cell loss after 6 months [45] and 35–36% cell loss after 12 months in the taco-folding technique [39].

On average, in our series, nDSAEK was performed 37.2 (±23.9 months) after primary MPK. This suggests that, although MPK grafts are generally expected to last at least 10–15 years, endothelial failure and graft-related complications can emerge postoperatively within the first months or years. This underscores the importance of regular and meticulous follow-up to detect early signs of graft dysfunction and ensure long-term surgical success.

No complications were observed during the procedure, and throughout the follow-up period, no immunological graft rejection or need for repeat DSAEK was observed. At the final follow-up visit, all grafts were functioning well, and no endothelial failure or rejection was reported. Wajima et al. conducted a study to compare the rejection rates following different types of keratoplasty. They found that the risk of rejection was lower after nDSAEK compared to PK but higher than after DMEK, similar to what has been observed with DSAEK. There was no significant difference in the risk of rejection between nDSAEK and DSAEK [46].

To our knowledge, this is the first study demonstrating the good visual outcomes and safety of DSAEK without Descemet membrane stripping to treat endothelial failure in primary MPK.

The main limitations of this study are the relatively small sample size and the short duration of the follow-up. The results in this small cohort are promising but may not be fully applicable to a broader population. The follow-up period in this study was limited to one year, which allowed for accurate assessment of visual and anatomical outcomes and monitoring of early complications. Still, it lacks information about long-term graft survival and complications. Although no significant sex-related differences in clinical outcomes were observed in our cohort, the limited sample size does not allow definitive conclusions. Future studies with larger populations and longer follow-up are warranted to investigate potential sex-based variations in response to nDSAEK after MPK. Additionally, alternative techniques such as DMEK, with and without Descemet membrane stripping, could be explored and compared to the procedure described in the study.

## 5. Conclusions

In conclusion, DSAEK without Descemet membrane stripping (nDSAEK) is a promising option for the surgical treatment of MPK endothelial failure, providing good visual and anatomical results. In this study, no cases of rejection and endothelial failure were observed, although the findings were limited by the relatively small sample size and the short follow-up period. Further research with larger patient cohorts and extended follow-up is needed to confirm these results and assess long-term efficacy and safety.

## Figures and Tables

**Table 1:** Patient demographics and surgical data.

Variable	Mean ± SD
Number of patients (n)	18
Age (mean years ± SD)	39.9 ± 13.5
Gender (Male/Female)	8 (44%)/10 (56%)
Indications for MPK:	
- Post-keratitis leucoma (n)	14 (77.7%)
- Traumatic corneal scar (n)	3 (16.7%)
- Advanced keratoconus (n)	1 (5.6%)
Interval between MPK and nDSAEK (mean months ± SD)	37.2 ± 23.9
Follow-up duration (mean months)	18.6 *

* Follow-up duration was at least 12 months in all patients.

**Table 2:** Preoperative vs. Postoperative Outcomes.

Variable	Preoperative (Mean ± SD)	Postoperative at 3 Months (Mean ± SD)	Postoperative at 6 Months (Mean ± SD)	Postoperative at 12 Months (Mean ± SD)	*p*-Value
BCVA (LogMAR mean ± SD)	1.40 ± 0.42	0.57 ± 0.23	0.48 ± 0.19	0.46 ± 0.19	<0.05
CCT (µm mean ± SD)	721 ± 70.12	624 ± 45.97	611 ± 50.43	616 ± 52.80	<0.05
ECD (cells/mm^2^ mean ± SD)	Not measurable	1809 ± 82	1780 ± 96	1748 ± 100	-

**Table 3:** Comparison of outcomes between patients who required re-bubbling and those who did not.

Variable	Total	Re-Bubbling	No Re-Bubbling
No. of patients (n)	18	2 (11.1%)	16 (88.9%)
BCVA at 12 months (logMAR mean ± SD)	0.46 ± 0.19	0.51 ± 0.27	0.46 ± 0.19
CCT at 12 months (µm mean ± SD)	616 ± 52.80	641 ± 26.16	613 ± 54.94
ECD at 12 months (cells/mm^2^ mean ± SD)	1748 ± 100	1612 ± 28	1765 ± 92

## Data Availability

The raw data supporting the conclusions of this article will be made available by the authors on request.
